# Efficacy of Intrauterine Lidocaine Instillation in Reducing Pain during Endometrial Biopsy by Novak

**DOI:** 10.1155/2018/9368298

**Published:** 2018-11-01

**Authors:** Sawanya Benchahong, Athita Chanthasenanont, Densak Pongrojpaw, Junya Pattaraarchachai, Kornkarn Bhamarapravatana, Komsun Suwannarurk

**Affiliations:** ^1^Department of Obstetrics and Gynecology, Faculty of Medicine, Thammasat University, Pathum Thani, Thailand; ^2^Chulabhorn International College of Medicine, Thammasat University, Pathum Thani, Thailand; ^3^Department of Preclinical Science, Faculty of Medicine, Thammasat University, Pathum Thani, Thailand

## Abstract

Abnormal uterine bleeding in women aged 35 years or over is an important clinical sign of many gynecological conditions. The diagnoses of these conditions require the pathological report of the endometrial tissue. Outpatient-based endometrial biopsy is an excellent option compared to standard fractional uterine curettage or hysteroscopy with endometrial biopsy in providing a definite diagnosis for abnormal uterine bleeding as it is less painful and does not require high potency anesthesia. This study evaluates the effect of intrauterine lidocaine on the patient's pain score during endometrial biopsy by the Novak curette. We included patients aged 35 years or more who had abnormal uterine bleeding between December 2016 and March 2018. The study was conducted at Thammasat University Hospital, Pathum Thani, Thailand. 250 patients were randomly allocated to either receive intrauterine lidocaine (study group) or normal saline (control group). Assessment of pain severity was evaluated using a visual analogue scale (VAS) score at 6 time-points, namely, before performing the procedure, when grasping the cervix by the tenaculum, during the intrauterine instillation of lidocaine or normal saline, during the uterine curettage, and then 15 minutes and 2 hours after the procedure. This study showed that there was significant pain reduction in patients who received intrauterine instillation of lidocaine compared to placebo, during uterine curettage, as well as 15 minutes and 2 hours after procedure (*p*<0.0001). Patient satisfaction was not significantly different between the two groups, while physician satisfaction significantly improved in the lidocaine group. Serious complications were not found during this study. (This research project had been approved for registration at Thai Clinical Trials Registry. TCTR identification number is TCTR20161031003.)

## 1. Introduction

Abnormal uterine bleeding in women aged 35 years or more is an important clinical sign of many gynecological problems such as endometrial hyperplasia, metritis, leiomyoma, endometriosis, and endometrial cancer. The gold standard for the diagnosis of abnormal uterine bleeding is endometrial histology. Endometrial tissue can be obtained from standard uterine curettage, office endometrial aspiration, or endometrial biopsy via hysteroscopy [1].

Outpatient-based endometrial biopsy is an excellent option compared to standard fractional uterine curettage or hysteroscopy with endometrial biopsy in providing a definite diagnosis for abnormal uterine bleeding. It is less painful and does not require high potency anesthesia [1], which itself can be associated with multiple complications such as paracervical or intracervical anesthetic infiltration.

Endometrial tissue aspiration devices are divided into two groups: plastic (low pressure) and metallic (high pressure) devices. Plastic devices available in Thailand include Pipelle (Unimar, CT, USA) and Endocelle (Wallach, CT, USA). Low pressure devices tend to produce less pain compared to high pressure devices [2]. However, there may be an issue of insufficient tissue sampling when using plastic devices [2].

The Novak endometrial curette is a high pressure endometrial biopsy device. It is a metallic cannula connected to a 10 ml syringe. Its diameter is larger than that of the plastic device and thus provides more pressure allowing a better tissue adequacy [2]. The Novak endometrial curette might therefore be a more suitable tool to ensure sufficient tissue sampling. However, the higher pressure can cause discomfort and pain to patients, thus reducing patient cooperation and satisfaction [2]. Therefore patients are likely to require some form of analgesia to minimize pain during this procedure.

Lidocaine, an aromatic benzene ring connected to an amide group, is a local anesthetic agent which inhibits the influx of sodium into the cell, thus preventing the occurrence of the neurotransmitter cascade. It has a rapid onset, short duration of action, low cost, minimal side effects, and good availability [3]. Recent literature showed that intrauterine lidocaine and bupivacaine instillation were appropriate methods for pain relief during intrauterine procedures [4–12].

In this study, we investigated the efficacy of intrauterine lidocaine during the endometrial biopsy in reducing pain and improving both patient and operator satisfaction.

## 2. Materials and Methods

This prospective double-blind randomized controlled trial was approved by the Ethics Committee, Faculty of Medicine, Thammasat University (MTU-EC-OB-2-132/59) (TCTR20161031003). The study was conducted at Thammasat University Hospital, Pathum Thani, Thailand. Patients aged 35 years or more who had abnormal uterine bleeding between December 2016 and March 2018 were included in this study. After individual counseling, the participants signed the informed consent. Exclusion criteria include patients with underlying coagulopathy, taking medicine involved with coagulation, allergy to lidocaine, pregnancy, pelvic inflammatory disease, and cervical stenosis, as well as those who decline to participate in the study. Patients' demographics data collected include age, body weight, height, body mass index (BMI), level of education, occupation, income, underlying diseases, hormonal usage, parity, vaginal delivery, history of uterine curettage, menopausal status, and indication for endometrial biopsy. Patients were randomly assigned into two groups using a computer generated table of random number. Group allocations were concealed in sealed opaque envelopes. Nurses at the outpatient department would open the envelopes once they met the participants.

For the procedure, each patient was placed in lithotomy position. The bivalve speculum and tenaculum were applied. The Novak curette connected to a syringe containing 7 ml of either lidocaine or normal saline solution (NSS) was inserted into the uterine cavity in the intervention or control group, respectively. After three minutes [8] of lidocaine instillation, endometrial sampling was performed by aspiration and rotation at 3, 6, 9, and 12 o'clock of uterine cavity. Assessment of pain severity was evaluated by using a visual analogue scale (VAS) recorded by the assisting nurse. The VAS score was ranked from score 0 (no pain) to 10 (worst pain). The result was then categorized into mild (0-4), moderate (5-6), or severe (7-10) pain. The patients' pain was evaluated at 6 different time-points, namely, before performing the procedure, when grasping the cervix by the tenaculum, during the intrauterine instillation of lidocaine or NSS, during the uterine curettage, and then 15 minutes and two hours after the procedure. Possible side effects, i.e., nausea, vomiting, vertigo, itching, and heavy bleeding per vagina were also recorded. All tissue samples were sent for pathological report.

The pathological reports of the endometrial tissue included the proliferative phase, secretory phase, atrophic or inactive endometrium, inadequate endometrial tissue, endometrial polyp, cancer, and others. The last category included benign endometrial tissue, acute inflammation, chronic inflammation, fragment of glandular, and stromal breakdown.

The sample size in this study was calculated from the standard deviation (SD) between study and control group from Guney's literature (SD = 1.46) [9]. The alpha and beta errors were set at 0.05, which suggested that the sample size should be at least 118 cases per group.

Data were analyzed by using the statistical package for social science (SPSS Inc, Chicago, IL USA) for Windows version 17. Continuous data were analyzed using the mean and unpaired t-tests. The Chi-square test was used for categorical data. Level of statistical significance was set at* p* value less than 0.05.

## 3. Results

**Figure 1:**
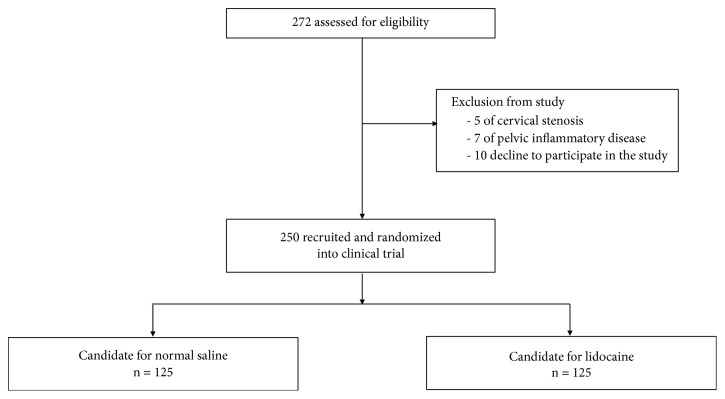
Participant flow diagram.

Out of 272 participants recruited, 250 met the inclusion criteria. Twenty-two patients were excluded from the study; 5 had cervical stenosis, 7 had pelvic inflammatory disease, and 10 declined to participate in the study. One hundred twenty-five participants were randomly assigned into either the control or the study group (Figure 1).

The patient's demographic data showed no statistical difference between the control and study groups (*p*>0.05 each) (Table 1). The minimum age was 35 years old in both groups. The maximum age was 77 years old in the study group and 73 years old in the control group, whereas the mean age was 48.5 and 47.2 years old, respectively. Around 60% of both groups (81 and 77 cases in study and control group) had previous vaginal delivery. Approximately one-third of the subjects had previous uterine curettage. There was no case that needed cervical dilatation. Indications for endometrial biopsy included menorrhagia, irregular cycle, intermenstrual bleeding, mixed symptoms, postmenopausal bleeding, and hypermenorrhea. There was no statistical difference between both groups (*p*=0.194).

**Figure 2:**
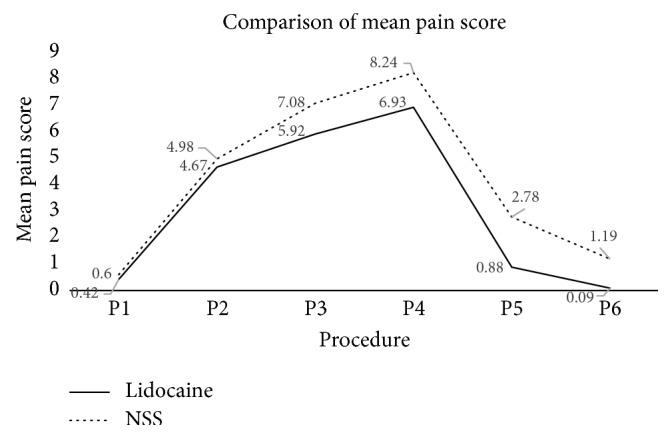
Comparison of mean pain score. P1: before procedure, P2: apply tenaculum, P3: during intrauterine infusion, P4: during aspiration, P5: 15 minutes after procedure, P6: 2 hours after procedure, NSS: normal saline.

In the control group, the VAS scores before performing the procedure, while grasping cervix by tenaculum, during intrauterine instillation, during uterine curettage, and 15 minutes and two hours after procedure were 0.60±1.40, 4.98±2.43, 7.08±2.23, 8.24±1.75, 2.78±2.49, and 1.19±1.79 (mean±SD), respectively, whereas the corresponding scores were 0.42±1.36, 4.67±2.38, 5.92±2.43, 6.93±2.19, 0.88±1.48, and 0.96±0.34 in the lidocaine group. There were significant pain reduction during intrauterine instillation, during uterine curettage, and 15 minutes and two hours after procedure in those who received intrauterine lidocaine compared to NSS (*p*<0.0001). The VAS pain scores before the procedure and while grasping cervix by tenaculum were not significantly different between the two groups (Table 2, Figure 2).

The mean duration of the procedure in study and control group were 5.12 and 5.40 minutes, respectively (*p*=0.13). The operative time varied from 3 to 10 minutes in the study and from 3 to 11 minutes in the control group.

Patient satisfaction was not significantly different between the two groups. Physician satisfaction was statistically superior in study group (Table 3).

Minimal cervical bleeding during tenaculum application was the only complication observed in this study. This was resolved by manual compression of the cervix with a gauze for 3-5 minutes. Serious complications, such as heavy vaginal bleeding, dizziness, and severe abdominal pain, were not found during this study.

There was no significant difference in pathological report in both groups. The two most common pathological results were proliferative phase of endometrium (24.4%) and secretory phase of endometrium (24%), respectively (Table 3). In this study, cancer prevalence was 3.2%, including one cervical cancer and 7 endometrial cancers. All cases of endometrial cancer presented with postmenopausal bleeding. The histopathological reports showed 6 endometrioid type and one clear cell type endometrial cancer. The patient with cervical cancer, however, presented with premenopausal abnormal vaginal bleeding. In this case it was the squamous cell carcinoma that was reported in the histopathology.

In patients with inadequate tissue sampling, fractional and curettage (F&C) of the endometrium was repeated. In this group, two out of 11 patients were lost during the follow-up. Six patients no longer had abnormal uterine bleeding and thus their status was changed to periodical observation. Only three patients underwent F&C. These three patients had no endometrial tissue.

## 4. Discussion

Endometrial biopsy has become an essential modality for obtaining the histological diagnosis of abnormal uterine bleeding [1]. It provides adequate endometrial tissue samples and can be easily performed in the gynecology outpatient departments. However, pain during the procedure may cause patients' discomfort and reduce compliance. Stovall and coworkers showed that endometrial sampling by the Pipelle caused less pain compared to the Novak, but also resulted in a higher number of insufficient tissue sampling [2]. Similarly, Kosus et al. reported that endometrial sampling was inadequate in 10% of cases when using the Pipelle curettage [4]. Moreover, the Pipelle is a single-use disposable device, whereas the Novak is nondisposable and can be sterilized for re-use during its lifetime. Therefore, the Novak is considered to be a more environmentally friendly, economical, and efficient endometrial sampling tool for the management of abnormal uterine bleeding.

Previous studies have investigated the efficacy of intrauterine lidocaine instillation for pain relief during endometrial aspiration. The studies of Kosus et al. [6], Dogan et al. [7], and Guler et al. [12] compared the intrauterine instillation of the lidocaine group to no anesthetic group while Maderak et al. [10] compared intrauterine lidocaine to sterile water during endometrial aspiration. Kosus, Trolice, and Sargin [4, 8, 11] also evaluated the efficacy of intrauterine lidocaine compared to normal saline in a similar fashion to this study. Similarly, this randomized, double-blind, controlled trial has shown that patients who received intrauterine lidocaine had significantly lower pain scores during the endometrial aspiration compared to the control group.

All previous studies used disposal plastic endometrial aspirators (Pipelle) [4, 6–12] while the present study used a metallic endometrial aspirator (Novak). We recruited a larger number of participants compared to previous studies and extended the evaluation of postoperative pain to 2 hours after the procedure. This is in contrast to previous studies [4, 6–12], which measured the patient's pain level during and up to thirty minutes after procedure. The analgesic effect of intrauterine lidocaine was still present at 15 minutes and 2 hours after the procedure. This may be explained by the inhibitory effects of lidocaine at the nerve endings located in the endometrial mucosa.

Effective analgesia during endometrial sampling is important for patient satisfaction and cooperation. In this study, although there was no statistically significant difference in patient satisfaction between the two groups, the physician satisfaction was significantly higher in the study group. This finding may be a result of improved patient compliance.

Although the Novak curette is rigid and has a larger diameter which may result in more discomfort, it has a better rate of adequate endometrial tissue sampling compared to other devices [2]. Insufficient endometrial sampling was present in only 4.4% in this study compared to 9.5% in Stovell's study [2].

In the present study, we found 8 cases of endometrial cancer. This further emphasizes the importance of adequate tissue sampling in abnormal uterine bleeding to prevent the misdiagnosis. The Novak curette used with intrauterine anesthesia serves as a good choice for obtaining the endometrial tissue.

## 5. Conclusion

Intrauterine lidocaine instillation during endometrial tissue sampling by the Novak curette significantly reduced patients' pain during intrauterine instillation, during uterine curettage, and 15 minutes and two hours after procedure. Cancer detection in this study was 3.2%. This showed that intrauterine lidocaine was an effective analgesia during endometrial sampling. The finding can help improve patient cooperation and operator satisfaction.

## Figures and Tables

**Table 1:** Demographic character of participants (n=125 each).

	Lidocaine	Normal saline	*p* value
Age (years) *∗*	48.53 ± 9.74	47.23 ± 8.29	0.26
BW (kg) *∗*	60.8 ± 11.34	61.82 ± 13.04	0.51
HT (cm) *∗*	157.26 ± 4.36	158.25 ± 5.26	0.11
BMI (kg/m^2^) *∗*	24.54 ± 4.20	24.65 ± 4.82	0.84
Education *∗∗*			0.64
Below primary level	26 (20.8)	27 (21.6)	
Secondary-tertiary level	52 (41.6)	54 (43.2)	
Above bachelor	47 (37.6)	44 (35.2)	
Occupation *∗∗*			0.28
House wife	22 (17.6)	26 (20.8)	
Agriculture	5 (4)	4 (3.2)	
SME	31 (24.8)	30 (24)	
Employee	43 (34.4)	43 (34.4)	
Government officer	24 (19.2)	22 (17.6)	
Income (Bath) *∗∗*			0.94
<10,000	15 (12)	18 (14.4)	
10,000 - 30,000	65 (52)	61 (48.8)	
30,000 - 50,000	31 (24.8)	32 (25.6)	
>50,000	14 (11.2)	14 (11.2)	
Underlying disease *∗∗*			
No	63 (50.4)	72 (57.6)	0.98
DM and HT	32 (25.6)	31 (24.8)	
Heart disease	1 (0.8)	4 (3.2)	
Anemia	2 (1.6)	2 (1.6)	
Other^a^	27 (21.6)	16 (12.8)	
Hormonal used *∗∗*			0.48
No	104 (83.2)	108 (86.4)	
Tamoxifen	3 (2.4)	3 (2.4)	
DMPA	7 (5.6)	5 (4)	
OCP	10 (8)	6 (4.8)	
Other^b^	1 (0.8)	3 (2.4)	
Parous cervix *∗∗*			0.6
Nulliparous	44 (35.2)	48 (38.4)	
Parous	81 (64.8)	77 (61.6)	
History of uterine curettage *∗∗*	31 (24.8)	31 (24.8)	0.79
Menopausal status *∗∗*			0.57
Premenopausal	93 (74.4)	89 (71.2)	
Postmenopausal	32 (25.6)	36 (28.8)	
Indication for endometrial biopsy *∗∗*			0.19
Menorrhagia	30 (24)	20 (16)	
Irregular cycle	23 (18.4)	34 (27.2)	
Intermenstrual bleeding	30 (24)	31 (24.8)	
Mixed symptoms	9 (7.2)	3 (2.4)	
Postmenopausal bleeding	32 (25.6)	36 (28.8)	
Hypermenorrhea	1 (0.8)	1 (0.8)	

*∗*: mean ± SD; *∗∗*: n (%); BW: body weight; HT: height; BMI: body mass index; SME: small and medium enterprise; DM: diabetes mellitus; HT: hypertension; Other^a^: breast cancer, asthma, thyroid disease, dyslipidemia, etc.; DMPA: depot medroxyprogesterone acetate; OCP: oral contraceptive pill; Other^b^: herb, progestin only pill, and implant.

**Table 2:** Comparison of pain score during endometrial aspiration (n=125 each).

Pain score at different stages	Lidocaine*∗∗*	Normal saline*∗∗*	*p* value
Before procedure			0.55
Mild	120 (96)	121 (96.8)	
Moderate	2 (1.6)	3 (2.4)	
Severe	3 (2.4)	1 (0.8)	
Apply tenaculum			0.53
Mild	54 (43.2)	49 (39.2)	
Moderate	40 (32)	37 (29.6)	
Severe	31 (24.8)	39 (31.2)	
During intrauterine infusion			0.01
Mild	29 (23.2)	14 (11.2)	
Moderate	40 (32)	31 (24.8)	
Severe	56 (44.8)	80 (64)	
During aspiration			<0.001
Mild	16 (12.8)	6 (4.8)	
Moderate	30 (24)	11 (8.8)	
Severe	79 (63.2)	108 (86.4)	
15 min after procedure			<0.001
Mild	117 (93.6)	93 (74.4)	
Moderate	7 (5.6)	18 (14.4)	
Severe	1 (0.8)	14 (11.2)	
2 hours after procedure			0.01
Mild	125 (100)	116 (92.8)	
Moderate	0	6 (4.8)	
Severe	0	3 (2.4)	

Before procedure: visual analogue scale (VAS) score before endometrial aspiration by Novak curette application; Apply tenaculum: VAS score during cervical grasping by tenaculum; During intrauterine infusion: VAS score during intrauterine lidocaine or normal saline infusion; During aspiration: VAS score during endometrial aspiration; 15 min after procedure: VAS score after 15 min of endometrial aspiration; 2 hours after procedure: VAS score after 2 hours of endometrial aspiration; *∗∗*: n (%); Mild: VAS score 0-4; Moderate: VAS score 5-6; Severe: VAS score 7-10.

**Table 3:** Pathological reports and satisfaction score (n=125 each).

	Lidocaine*∗∗*	Normal saline*∗∗*	*p* value
Pathological reports			0.8
Proliferative phase	26 (20.8)	35 (28)	
Secretory phase	29 (23.2)	31 (24.8)	
Atrophic or inactive ET	31 (24.8)	26 (20.8)	
No or inadequate ET	7 (5.6)	4 (3.2)	
Endometrial polyp	13 (10.4)	10 (8)	
Cancer	4 (3.2)	4 (3.2)	
Other	15 (12)	15 (12)	
Satisfaction score			
Patient's*∗*	9.00 ± 1.35	8.86 ± 1.35	0.4
Physician's*∗*	9.27 ± 0.786	8.74 ± 1.37	< 0.001

*∗∗*: n (%), ET: endometrial tissue, Other: benign endometrial tissue, acute inflammation, chronic inflammation and fragment of glandular and stromal breakdown, *∗*: mean ± SD.

## Data Availability

The raw data used to support the findings of this study were supplied by Dr. Chanthasenanont under license and so cannot be made freely available. They are restricted by the ethics board of Faculty of Medicine, Thammasat University, Pathumthani, Thailand, in order to protect the patients' privacy. Requests for access to these data should be made to Dr. Benchahong by researchers who meet the criteria for access to confidential data.
